# Robotic Horizons in Plastic Surgery: A Look Toward the Future

**DOI:** 10.3390/jcm15020602

**Published:** 2026-01-12

**Authors:** Ali Foroutan, Diwakar Phuyal, Georgia Babb, Julia Ting, Ghazal Mashhadiagha, Niayesh Najafi, Risal Djohan, Sarah N. Bishop, Graham S. Schwarz

**Affiliations:** 1 Department of Plastic Surgery, Cleveland Clinic, 9500 Euclid Avenue, A60, Cleveland, OH 44195, USA; ali.foroutan@uhhospitals.org (A.F.); phuyald@ccf.org (D.P.); bishops@ccf.org (S.N.B.); 2 Department of Applied Data Science, Cleveland State University, Cleveland, OH 44115, USA; 3 College of Osteopathic Medicine, Pacific Western University of Health Sciences, Pomona, CA 91766, USA; niayesh.najafi@westernu.edu

**Keywords:** robotic surgery, plastic surgery, microsurgery, breast reconstruction, lymphatic surgery

## Abstract

**Background/Objectives:** Robotic technology has transformed several surgical specialties, offering enhanced precision, visualization, and dexterity. In plastic and reconstructive surgery, robotic systems are increasingly utilized across a range of procedures, though their applications remain in early development. **Methods:** A review of the literature was performed to identify studies reporting robot-assisted procedures in plastic and reconstructive surgery. The literature was synthesized thematically to characterize current procedural applications, emerging technologies, and areas of active clinical investigation. **Results:** Robotic systems have been reported in a broad range of plastic and reconstructive procedures, including flap harvest, microsurgery, breast reconstruction, craniofacial and head and neck reconstruction, esthetic surgery, and gender-affirming surgery. The existing studies primarily consist of case series and case reports with substantial variability in reported indications, techniques, and technological platforms. Comparative clinical outcomes and long-term data are limited. **Conclusions:** Robot-assisted reconstruction continues to expand across multiple procedural domains. However, current evidence remains largely descriptive, underscoring the need for standardized reporting and prospective studies to better define clinical value, safety, and appropriate indications.

## 1. Introduction

Robot-assisted surgery represents one of the most significant technological advancements in modern surgical practice [1]. Plastic and reconstructive surgery inherently demands exceptional precision, fine motor control, and three-dimensional spatial awareness to address intricate and variable anatomical structures. The integration of robotic systems offers an opportunity to enhance these capabilities, providing improved visual-spatial resolution, instrument dexterity, tremor reduction, and motion scaling beyond the limitations of human performance [2].

While robotic technology has been widely adopted in urologic, gynecologic, and cardiothoracic surgery, its incorporation into plastic and reconstructive surgery remains limited but is rapidly expanding [2]. Early applications, ranging from transoral reconstruction of oropharyngeal defects to muscle and perforator flap harvest, and more recent developments in microvascular anastomosis, demonstrate the feasibility of robotic approaches in procedures requiring delicate tissue handling and precise dissection within confined spaces [2,3,4].

The advantages of robotic platforms extend beyond technical precision. They enable smaller incisions, reduced blood loss, and diminished donor-site morbidity, contributing to improved recovery and esthetic outcomes [2,3]. From the surgeon’s perspective, robotic platforms may facilitate broader adoption of complex technical procedures such as super microsurgery [5], improve ergonomics through motion stabilization, reduce fatigue, and enhance performance during prolonged procedures [2]. Moreover, the integration of robotic connectivity and artificial intelligence enables opportunities for remote collaboration, telesurgery, quality benchmarking, and standardized training across institutions—advancing access to specialized care, reducing regional disparities, and promoting equity in surgical outcomes [6,7].

Despite these potential advantages, robotic applications in plastic and reconstructive surgery remain distributed across diverse procedural domains, and the existing literature is largely fragmented and predominantly composed of case reports and small case series. To date, no single publication has comprehensively synthesized the full scope of robotic applications across the specialty. This review aims to critically evaluate the current state of robotic technology in plastic and reconstructive surgery, delineate existing limitations and barriers to implementation, and propose a framework for its clinical integration and technological advancement.

## 2. Methods

This perspective is informed by a targeted, non-systematic review of the contemporary literature on robotic applications in plastic and reconstructive surgery. Representative publications were identified through focused searches of PubMed, Scopus, and Web of Science using combinations of the terms “robotics,” “robotic surgery,” “plastic surgery,” and “reconstructive surgery”. The final search was completed in October 2025. Publications were selected based on relevance to robotic assistance in reconstructive, esthetic, microsurgical, craniofacial, lymphatic, and gender-affirming procedures, as well as their contribution to illustrating current practice patterns and emerging technical directions. Emphasis was placed on studies that described operative techniques, workflows, or early clinical implementation of robotic platforms. The literature included comprises case reports, case series, cohort studies, feasibility studies, and early clinical trials that described robotic techniques, workflows, or applications relevant to plastic and reconstructive surgery. Purely educational or training-focused studies, studies centered on non-plastic or non-reconstructive surgical specialties, non-procedural publications (including editorials, commentaries, and opinion pieces), and animal-only or preclinical studies were excluded from analysis. Simplified PRISMA and search strategy are listed in the Supplementary Materials document.

## 3. Current Applications in Plastic Surgery

Robotic technology has been applied across a broad spectrum of plastic and reconstructive surgical procedures, particularly in settings where enhanced visualization, precision, and access to confined anatomical spaces may be advantageous. Reported applications include flap harvest (such as Deep Inferior Epigastric Perforator (DIEP), latissimus dorsi, rectus abdominis, and omental flaps), microsurgical and super microsurgical procedures (including free flap anastomosis, lymphaticovenular anastomosis, and nerve coaptation), implant-based breast reconstruction, craniofacial and head and neck reconstruction (notably transoral robotic surgery-assisted reconstruction), esthetic procedures, and gender-affirming surgery (including robotic-assisted vaginoplasty and peritoneal flap harvest). Across these domains, the existing majority of the literature consists of case reports and case series with only limited high-quality comparative studies, reflecting the early and evolving nature of the field.

### 3.1. Flap Harvest

Robotic platforms can assist with the precise dissection of pedicle vessels during flap harvest, potentially reducing the need for larger incisions, muscle transection, or denervation (Table 1). This is particularly relevant in procedures such as DIEP flap harvest, where open approaches may require partial transection or denervation of the rectus abdominis muscle to access the deep inferior epigastric artery [8]. The purported promise of the DIEP flap technique has been preservation of rectus abdominis function, yet the most common long-term donor site morbidity following DIEP flap harvest is abdominal bulging due to this dissection, with reported rates ranging from 2 to 33% [8,9]. Two approaches have been described for robotic harvest of the DIEP flap. The multi-port robotic transabdominal pre-peritoneal (TAPP) was first performed by Gundlapalli et al. in 2018 [10]. In the multi-port robot-assisted totally extraperitoneal (TEP) reconstruction, which was first described in a cadaver model by Manrique et al. in 2019, and performed clinically by Bishop and Schwarz, the plane of dissection is between the rectus abdominis muscle and the posterior rectus sheath [11,12]. The TEP technique allows avoidance of intra-abdominal entry, although due to the narrow working space, it might have a steeper learning curve compared with the TAPP technique [11,13]. A single-port robotic system has also been reported for DIEP flap dissection using the TEP approach in unilateral cases by Lee et al. [13]. Although no definitive advantage has been demonstrated between the TEP and TAPP approaches, the existing literature supports the feasibility of both techniques, with approach selection largely dependent on surgeon experience and comfort with robotic assistance.

Similarly, robotic assistance may be beneficial in harvesting flaps such as the latissimus dorsi or rectus abdominis muscle, where minimizing donor-site morbidity and scar burden is desirable [14,15]. Selber et al. demonstrated the feasibility of robotic latissimus dorsi flap harvesting [16]. The potential advantages of robotic harvest may apply to both muscle-only flaps and musculocutaneous flaps with a small skin paddle.

From a technical standpoint, robotic flap harvest requires careful planning of multi-port placement and instrument selection to optimize exposure while minimizing tissue trauma. For DIEP flaps, ports are typically positioned to provide a direct trajectory to the deep inferior epigastric vessels, allowing precise intramuscular dissection and perforator preservation [3,12,17]. The robotic arms facilitate gentle retraction and separation of tissue layers. Single-port techniques can be especially advantageous in confined spaces, allowing simultaneous dissection and flap mobilization without additional incisions. Additionally, the surgeon can dynamically adjust camera angles and magnification through the console, ensuring continuous visualization of critical landmarks throughout the procedure. A single port approach has shown promise, particularly in the latissimus dorsi flap harvest [18], but can also be utilized in the DIEP flap harvest [13]. Early reports suggest that robotic harvest may lead to improved postoperative outcomes, particularly in terms of pain control and reduced length of hospital stay [8,19,20].

The omental flap is a versatile reconstructive option with a wide range of applications, including lymphedema. Traditionally, harvesting this flap has required a laparotomy. More recently, both multi-port and single-port robotic approaches have been employed [21], offering enhanced visualization, reduced donor-site morbidity, and improved postoperative recovery [22].

Although the current literature remains largely limited to small case series and case reports, the number of published experiences continues to increase annually. As larger cohorts and prospective studies emerge, the true added value of robotic flap harvest—particularly with respect to outcomes such as donor-site morbidity, bulge or hernia rates, and length of hospital stay—will be more definitively characterized.

Cost remains a frequently cited barrier to the adoption of robotic techniques. While the upfront capital investment of robotic platforms is substantial, the per-case cost of robotic utilization may be more nuanced and institution-dependent. As previously discussed by Selber, the primary determinant of cost-effectiveness is not the acquisition cost of the robotic platform itself, but rather the contribution margin per case, which reflects the balance between procedural revenue and incremental costs related to disposables, staffing, and operative time [3]. In institutions where robotic infrastructure is already established and utilization is sufficient, these incremental costs may be partially offset by potential downstream benefits, including reduced length of stay, lower donor-site morbidity, and enhanced recovery. However, robust cost-effectiveness analyses specific to robotic flap harvest remain limited, and prospective comparative studies are necessary to determine whether observed clinical advantages translate into meaningful economic benefit.

**Table 1:** Representative studies describing robot-assisted flap harvests and reconstructions are reported in the literature.

S.N	Author, Year	Study Design	LOE	Country	Procedure/Flap Type	Number of Patients	Indication	Robot Used	Application/Role of Robot
1	Selber et al., 2012 [16]	Cadaveric Study	N/A	USA	Latissimus dorsi flap	10	Breast reconstruction	Da Vinci S	Flap harvest
2	Clemens et al., 2014	Retrospective chart review	3	USA	Latissimus dorsi flap	146	Breast reconstruction	Da Vinci	Flap harvest
3	Chung et al., 2015	Case series	4	Korea	Latissimus dorsi flap	12	Breast reconstruction	Da Vinci S	Flap harvest
4	Gundlapalli et al., 2018 [10]	Case Report	5	USA	TAPP DIEP	1	Breast reconstruction	Da Vinci	Flap harvest
5	Benjoar et al., 2018	Case Report	5	USA	TEP DIEP	1	Breast reconstruction	Da Vinci SI	Flap harvest
6	Shakir et al., 2020	Cohort	3	USA	TAPP DIEP	3	Breast reconstruction	Da Vinci XI	Flap harvest
7	Winocour et al., 2020	Cohort	3	USA	Latissimus dorsi flap	25	Breast reconstruction	Da Vinci	Flap harvest
8	Daar et al., 2021	Case Series	4	USA	TAPP DIEP	4	Breast reconstruction	Da Vinci XI	Flap harvest
9	Day et al., 2021	Case Report	5	USA	Omentum flap	1	Breast reconstruction	Da Vinci	Flap harvest
10	Joo et al., 2021	Case report	5	Korea	Latissimus dorsi flap	1	Breast reconstruction	Da Vinci SP	Flap harvest
11	Wittesaele and Vandervoort, 2022	Case Series	4	Belgium	TAPP DIEP	10	Breast reconstruction	Da Vinci	Flap harvest
12	Bishop et al., 2022 [19]	Case Series	4	USA	TAPP DIEP	21	Breast reconstruction	Da Vinci	Flap harvest
13	Lee et al., 2022 [13]	Cohort	3	Korea	TEP DIEP	21	Breast reconstruction	Da Vinci SP	Flap harvest
14	Cheon et al., 2022 [14]	Case Series	4	Korea	Latissimus dorsi flap	41	Breast reconstruction	Da Vinci Si, Da Vinci Xi, Da Vinci SP	Flap harvest
15	Shuck et al., 2022	Cohort	3	USA	Latissimus dorsi flap	15	Breast reconstruction	Da Vinci	Flap harvest
16	Dayaratna et al., 2022	Case Report	5	Australia	TAPP DIEP	1	Breast reconstruction	Da Vinci Xi	Flap harvest
17	Hwang et al., 2022 [18]	Case series	4	Korea	Latissimus dorsi flap	3	Breast reconstruction	Da Vinci SP	Flap harvest
18	Jung et al. 2022	Case report	5	Korea	TEP DIEP	1	Breast reconstruction	Da Vinci SP	Flap harvest
19	Tsai et al., 2023 [20]	Cohort	3	Taiwan	TAPP DIEP	13	Breast reconstruction	Da Vinci XI	Flap harvest
20	Moreira et al., 2024	Cohort	3	USA	TAPP DIEP	23	Breast reconstruction	Da Vinci X/XI	Flap harvest
21	Kim et al., 2024	Cohort	3	Korea	DIEP/NSM	153 (rNSM), 64 (rDIEP)	Breast reconstruction	Da Vinci SP	Flap harvest/Mastectomy
22	Phuyal et al., 2025 [8]	Case Report	5	USA	TAPP DIEP	1	Breast reconstruction	Da Vinci Xi	Flap harvest
23	Kuo et al., 2025	Cohort	3	Taiwan	DIEP/mastectomy	14	Breast reconstruction	Da Vinci Xi	Flap harvest/Mastectomy
24	Bishop et al., 2025 [12]	Retrospective review	3	USA	Unilateral TEP DIEP	NR	Breast Reconstruction	Da Vinci Xi	Flap harvest/mastectomy
	**Other Indications**								
1	Patel et al., 2011	Case report	5	USA	Latissimus dorsi flap	1	Shoulder reconstruction	Da Vinci	Flap Harvest
2	Patel and Pedersen, 2012	Case Report and preclinical study	5	USA	Rectus abdominis muscle	1	Lower extremity reconstruction	Da Vinci	Flap Harvest
3	Pedersen et al., 2014 [15]	Cohort Study	3	USA	Rectus abdominis muscle	10	Pelvic reconstruction	Da Vinci	Flap Harvest
4	Ciudad et al., 2016	Case Report	5	Korea	Gastroepiploic lymph node flap	1	Lymphedema	Da Vinci	Flap dissection and inset
5	Ozkan et al., 2019	Case Report	5	Turkey	Omentum flap	1	Lower extremity reconstruction	Da Vinci	Flap Harvest
6	Moon et al., 2020	Cohort Study	3	South Korea	Latissimus dorsi flap	21	Poland syndrome/chest wall reconstruction	Da Vinci	Flap Harvest
7	Fouarge et al., 2020	Case series	4	Belgium	Latissimus dorsi flap	6	Upper/lower limb reconstruction	Da Vinci Xi, Da Vinci SP	Flap harvest
8	Frey et al., 2020 [22]	Case series	4	USA	Omentum flap	5	Vascularized lymph node transfer for upper extremity lymphedema	Da Vinci SP	Flap harvest
9	Teven et al., 2021	Case Report	5	USA	VOLT	1	Lymphedema	Da Vinci SP	omental harvest
10	Asaad et al., 2021	Case series	4	USA	Rectus abdominis flap	7	Pelvis reconstruction	NR	Muscle harvest
11	Haverland et al., 2021	Case series	4	USA	Rectus abdominis flap	6	Vesicovaginal fistula, complex pelvic organ prolapses, anterior and posterior exenteration, partial and total vaginectomy, partial vulvectomy, and abdominoperineal resection.	NR	Flap harvest
12	Armando et al., 2022	Cohort Study	3	USA	Rectus abdominis flap	36	Pelvic reconstruction	Da Vinci	Flap harvest
13	Sanchez-Rodriguez et al., 2024	Case Report	5	Spain	Omentum flap	1	Lymphedema	Da Vinci Xi	Omental Dissection
14	Iftekhar et al., 2025	Retrospective review	3	USA	Rectus abdominis flap	32	posterior vaginal wall reconstruction	Da Vinci	Flap harvest

S.N—serial number; SGA—superior gluteal artery; SIEA—superficial inferior epigastric artery; SGAP—superior gluteal artery perforator; TAPP—transabdominal preperitoneal approach; TEP—totally extraperitoneal approach; DIEP—deep inferior epigastric perforator; PAP—profunda artery perforator; NSM—nipple-sparing mastectomy; rNSM—robotic nipple-sparing mastectomy; rDIEP—robotic deep inferior epigastric perforator flap; VOLT—Vascularized omentum lymphatic transplant; NR—not reported, N/A: not applicable, LOE—American Society of Plastic Surgery Level of Evidence.

### 3.2. Robotic Breast Reconstruction with Prosthesis

Although the clinical utility and effectiveness of robot-assisted mastectomy and breast reconstruction remain subjects of ongoing debate, several reports describing these techniques have been published (Table 2) [23,24]. Prosthesis-based robot-assisted breast reconstruction has been reported with the use of small lateral incisions, offering the potential for reduced visible scarring. Both Da Vinci SP and XI robots have been utilized for this purpose [24]. It has been hypothesized that carbon dioxide insufflation during robotic dissection, particularly with single-port systems, may be less traumatic to mastectomy skin flaps than manual retraction, potentially reducing the risk of flap ischemia or necrosis. This potential benefit was suggested by Kim et al. in a cohort study; however, further comparative studies are required to substantiate these findings [24].

Although prosthesis reconstructions are technically feasible, careful attention must be paid to the preservation of key esthetic landmarks during reconstruction. In particular, maintaining the integrity of the inframammary fold may be challenging in the robotic setting, where tactile feedback and traditional visual cues are altered. Additionally, deliberate efforts are required to preserve the midline to avoid inadvertent obliteration, which may predispose to symmastia.

### 3.3. Robotic Microscope

There are several technologies available for surgical field magnification, including surgical loupes, operative microscopes, and exoscopes. Although the operative microscope remains the most widely used tool, it can be cumbersome, requiring repeated manual adjustments, and it can be ergonomically challenging during prolonged procedures. In response to these limitations, robotic digital microscopes-such as the RoboticScope (BHS Technologies, Innsbruck, Austria), were introduced, integrating high-resolution three-dimensional visualization with virtual reality (VR) interfaces (Table 3) [25,26]. These platforms enable hands-free, head gesture-controlled navigation of the surgical camera, allowing surgeons to adjust the operative view without breaking sterility or disrupting workflow. While early experience suggests potential ergonomic advantages compared with conventional microscopes, further objective evaluation is needed to determine their impact on surgeon fatigue, efficiency, and clinical outcomes.

Moreover, the combination of image stabilization with magnified three-dimensional visualization may be particularly relevant for technically demanding microsurgical tasks, including vascular anastomosis, free flap dissection, and lymphaticovenular anastomosis. As these technologies continue to evolve, robotic digital visualization systems may also play a role in surgical education and tele-mentoring by enabling real-time, shared, virtual reality-based operative views, although their broader clinical and educational impact remains to be defined [25,26].

### 3.4. Microsurgery

Robotic systems such as MUSA (Microsure, Eindhoven, The Netherlands) and Symani (MMI, Pisa, Italy) have been developed to facilitate high-precision microsurgical procedures (Table 4) [4,27]. Reported applications include lymphovenous anastomosis, free flap microvascular anastomosis, and nerve coaptation [4,27]. The platforms are teleoperated, with the surgeon seated and viewing the operative field through an exoscope or robotic digital microscope rather than a conventional microscope [4]. The surgeon’s movements are translated to wristed microinstruments (Symani, MMI, Pisa, Italy) with multiple degrees of freedom, enabling fine manipulation at a submillimeter scale. While this configuration may reduce physical strain, its impact on operative efficiency and outcomes remains under evaluation [4].

Micro-robotic systems may be particularly relevant in super microsurgery, where vessel diameters are often less than 0.8 mm and technical precision approaches the limits of human dexterity [4,27]. These systems incorporate motion scaling, tremor filtration, and console-based operation, which may assist surgeons when working with delicate structures. These technologies also hold promise for extending the operative careers of microsurgeons by reducing physical strain, improving posture, and mitigating the effects of age-related physiologic tremor. In addition, microneural surgery-including procedures on the brachial plexus-has emerged as another promising field for robotic assistance, offering improved visualization and fine motor control during intricate nerve repairs.

Comparative studies have suggested that robotic assistance with platforms such as Symani may reduce vessel edge trauma during anastomosis compared with conventional techniques [28,29,30]. Surgeons also reported improved scores in intraoperative tremor suppression, reduced muscle fatigue, enhanced optical detail, greater operative comfort, and superior depth and 3D structural visualization when using robotic systems. However, Jeong et al. reported longer anastomotic times and steep learning curves with the Symani. High rates of intraoperative anastomosis patency (>90%) were reported but remain to be evaluated in head-to-head comparisons with conventional methods [28]. Furthermore, the performance of manual super microsurgery engenders limited haptic feedback at baseline, making its absence relatively inconsequential for surgeons who routinely perform these procedures [28,29,30].

From a practical standpoint, the clinical introduction of microrobotic systems such as MUSA and Symani requires adjustments in operative workflow and training. Although setup is generally achievable within minutes, it requires appropriate preoperative planning and team familiarity. Current platforms are compatible with standard operating rooms and utilize compact robotic arms positioned on either side of the patient. Surgeons typically undergo dedicated training sessions, including dry-lab simulations on synthetic vessels and animal models, before progressing to clinical cases. Early experience suggests that the learning curve for most microsurgeons adapting to motion scaling and wristed microinstruments occurs within 10–20 practice cases [28]. As clinical experience expands, broader adoption of micro-robotic systems will depend on practical considerations such as cost, availability of instruments, training requirements, and seamless integration with visualization platforms. Further comparative and long-term studies will be necessary to clarify whether these technologies translate into consistent clinical or ergonomic advantages over conventional microsurgical techniques.

**Table 4:** Representative studies describing robot-assisted microsurgery and free flap reconstruction in plastic and reconstructive surgery. LOE: American Society of Plastic Surgery Level of Evidence.

S.N	Author, Year	Study Design	LOE	Country	Procedure/Flap Type	Number of Patients	Indication	Robot Used	Application/Role of Robot
1	Boyd et al., 2006	Case series	4	USA	Muscle-sparing TRAM flap (11), SGA flap (6), SIEA flap (4), and SGAP flap (1)	20	Breast reconstruction	Aesop	Internal Mamary Artery Dissection
2	Barbon et al., 2022	Case Series	4	Switzerland	PAP, gracilis neurovascular flap, SCIP	22	Lymphatic reconstructive surgery (18), free flap reconstruction (3), nerve coaptation (1)	Symani	Robot-assisted anastomosis
3	van Mulken et al., 2022	Cohort Study	3	The Netherlands	LVA	8	Lymphedema	MicroSure MUSA	Robot-assisted anastomosis
4	Lindenblatt et al., 2022	Cohort Study	3	Switzerland	LVA/free vascularized lymph node transfer	5	Lymphedema	Symani	Robot-assisted anastomosis
5	Beier et al., 2023	Case Series	4	Germany	Radial forearm flap (11), ALT-flap (7), fibular flap (4), and anterior serrate muscle flap (1)	23	Free flap reconstruction (various)	Symani	Robot-assisted anastomosis
6	Besmens et al., 2023	Case Series	4	Switzerland	medial femoral condyle free flap (2), ALT free flap (1), lateral arm free flap (1), nerve grafting with nerve allograft (2)	6	Arterial anastomoses (4), nerve grafting (2)	Symani	Robot-assisted anastomosis
7	Schafer et al., 2023	Case report	5	Germany	Nerve transfer/Intercostal nerves to long thoracic and thoracodorsal nerve	1	Brachial Plexus Palsy	Symani	Nerve transfer
8	Grunherz et al., 2023	Case report	5	Switzerland	Central lymphatic reconstruction	1	Central lymphatic dilation	Symani	Microsurgical anastomoses
9	Innocenti et al., 2023	Case report	5	Italy	ALT	1	Post traumatic	Symani	Microsurgical anastomoses
10	Martin et al., 2024	Case Series	4	Germany	Peripheral nerve surgery	19	Nerve transfer, muscle reinnervation, neurotized free flaps, autologous nerve grafts	Symani	Nerve coaptation
11	Reibnitz et al., 2024	Cohort Study	3	Switzerland	LTT/LVA/LLA	67	Lymphedema	Symani	Robot-assisted anastomosis
12	Tolksdorf et al., 2024	Cohort Study	3	Germany	Radial forearm, fibula, latissimus dorsi, scapula	30	Cranio- and maxillofacial surgery	Symani	Robot-assisted anastomosis
13	Struebing et al., 2024	Cohort Study	3	Germany	Free flap (ALT, latissimis dorsi, DIEP, etc.), Nerve surgery, LVA (Various)	100	Various	Symani	Various
14	Struebing et al., 2024	Cohort Study	3	Germany	ALT, medial femoral condyle, latissimus dorsi	16	Upper extremity defects	Symani	Robot-assisted anastomosis
15	Dastagir et al., 2024	Retrospective chart review	3	Germany	Finger replantation (8), finger blood vessel injury (13)	21	Hand reconstruction	Symani	Robot-assisted anastomosis
16	Reilly et al., 2024 [29]	Cohort Study	3	Sweden	LVA	12	Lymphedema	MUSA-2	Robot-assisted anastomosis
17	Mori et al., 2024	Cohort Study	3	Italy	ALT (5), medial plantar (1), SCIP (1), latissimus dorsi (2), serratus anterior (1), medical femoral condyle (1), free fibular (3), free toe pulp (1), sensate free-style perforator flap from ulnar artery (1)	16	Various	Symani	Robot-assisted anastomosis
18	Lilja, et al., 2024	Case Report	5	Denmark	LVA/lymphocele excision	1	Lymphocele	Symani	Robot-assisted anastomosis, excision
19	Gorji et al., 2024	Retrospective review	3	Germany	DIEP (10), ALT (4), gracilis (4), SCIP (2), PAP (2), latissimus dorsi (1)	23	Cancer, posttraumatic	Symani robot, RoboticScope microscope	Microsurgical anastomoses
20	Vollbach et al., 2024	Case Report	5	Germany	DIEP	1	Breast Reconstruction	Symani	Robot-assisted anastomosis
21	Watson et al., 2025	Cohort Study	5	Switzerland	Free ALT or latissimus dorsi to scalp/facial artery and vein	6	Free tissue transfers for defects of the scalp	Symani	Robot-assisted anastomosis
22	Chen et al., 2025 [17]	Case Series	4	USA	Lymph node-to-vein anastomosis	20	Lymphedema	Symani	Robot-assisted anastomosis
23	Spille et al., 2025	Cohort Study	3	Germany	Radial forearm flap, ulnar forearm, fibula	93	Head and neck reconstruction	Symani	Robot-assisted anastomosis
24	Sorensen et al., 2025	Cohort Study	3	Denmark	ALT, DIEP, fibular, helical and LVA	12	Various	Symani	robot-assisted anastomosis
25	Paternoster et al., 2025	Case report	5	UK	DIEP	1	Chest wall reconstruction	Symani	robot-assisted anastomosis
26	Kukreja-Pandey et al., 2025	Case report	5	USA	LVB	1	Breast lymphedema	Symani	LVB
27	Konneker et al., 2025	Cohort Study	3	Switzerland	SCIP (2), ALT (6)	8	Upper and lower extremity reconstruction	Symani	Robot-assisted anastomosis

S.N—serial number; TRAM—transverse rectus abdominis musculocutaneous; SGA—superior gluteal artery; SIEA—superficial inferior epigastric artery; SGAP—superior gluteal artery perforator; PAP—profunda artery perforator; ALT—anterolateral thigh; DIEP—deep inferior epigastric perforator; LVA—lymphaticovenular anastomosis; LTT—lymphatic transfer; LLA—lymphatic-lymphatic anastomosis; SCIP—superficial circumflex iliac artery perforator; LVB—lymphatic-venous bypass; LOE—American Society of Plastic Surgery Level of Evidence. Numbers in brackets indicate the total number of flaps for each procedure.

### 3.5. Head and Neck/Craniofacial Reconstruction

Before the introduction of Trans-Oral Robotic Surgery (TORS), surgical management of oropharyngeal and hypopharyngeal tumors often required highly morbid open approaches, such as mandibulotomy or lip-splitting incisions, which carried substantial risks of cosmetic deformity, prolonged recovery, and impaired swallowing and speech. Even with transoral laser microsurgery, visualization and maneuverability in deep anatomical corridors remained limited. The advent of robotic surgery addressed some of these challenges by enabling access to narrow spaces through natural orifices, improving visualization, and enhancing surgeon ergonomics, thereby facilitating minimally invasive tumor resection.

TORS was pioneered in the mid-2000s by Drs. Bert O’Malley Jr. and Gregory Weinstein at the University of Pennsylvania, who demonstrated its clinical benefits with minimally invasive approaches to oropharyngeal lesions—first in preclinical models and later in patients—with the term officially entering the surgical field in 2005 [31]. Following early clinical experience, the U.S. Food and Drug Administration approved the Da Vinci system to perform TORS on benign and early-stage malignant head and neck tumors in December 2009 (Table 5). Since then, TORS has been increasingly adopted for appropriately selected cases, supported by its ability to provide three-dimensional visualization, wristed instrumentation, tremor reduction, and precise access to confined anatomical spaces.

Beyond its initial application in tonsillar and base-of-tongue tumors, TORS has been extended to lesions of the hypopharynx, parapharyngeal space, supraglottic larynx, and carcinoma of unknown primary, where enhanced visualization may facilitate identification of occult disease in accordance with current guidelines. TORS has also been explored in the management of obstructive sleep apnea in patients resistant to conventional therapies [35].

More recently, robotic platforms have been applied in reconstructive and craniofacial surgery, including TORS-assisted flap inset, selected free flap reconstructions, and investigational applications in hemifacial microsomia, genioplasty, Le Fort osteotomies, and mandibular contouring (Table 5) [32,33,34]. Overall, robotic use in craniofacial surgery spans oncologic resection, flap inset, cleft palate repair, and osteotomy. However, broader adoption remains limited by the paucity of high-quality evidence, as most published data consist of cohort studies, case reports, and small case series.

### 3.6. Esthetic Procedures

Robotic technology is in its early stages within facial rejuvenation but shows considerable promise [36]. Surgical robots have been reported to enhance precision in the dissection and visualization of delicate facial structures, offering up to twenty times magnification and tremor-free movement [36]. The Da Vinci system has been explored for esthetic face and neck procedures, rectus diastasis repair with abdominal body contouring, although early experiences required innovative docking strategies to accommodate the constraints of the robotic arms [36].

Hair restoration is currently the most developed use of robotics in esthetics. Robotic follicular unit extraction systems like ARTAS (Venus Concept, Toronto, ON, Canada) and NeoGraft (Venus Concept, Toronto, ON, Canada) have been in use for over a decade [37,38]. ARTAS, FDA-approved in 2011, uses image-guided algorithms to identify and harvest follicular units with precision. Advantages include consistent graft quality, reduced surgeon fatigue, and precise targeting, although limitations include cost, slower speed compared to skilled manual teams, and reduced accuracy with certain hair types.

Robotic assistance is slowly expanding beyond hair and facial applications into other fields of esthetic surgery (Table 6). Uptake is slower than in other specialties due to the artistic and individualized nature of cosmetic work [39]. Continued refinement in artificial intelligence (AI) guidance, force feedback, and specialized instruments may allow robots to enhance surgical artistry while maintaining safety and patient confidence.

### 3.7. Gender-Affirming Surgery

Robot-assisted vaginoplasty has evolved rapidly over the past decade, transitioning from isolated case reports to large multi-institutional series (Table 7). Early reports, such as Boztosun and Olgan (2016) [40], described the use of the Da Vinci Xi system for sigmoid vaginoplasty in Mayer–Rokitansky–Küster–Hauser syndrome, while subsequent studies demonstrated its application in gender-affirming surgery for flap dissection, harvest, and inset [40,41]. Between 2019 and 2025, multiple case series have documented increasing procedural volume and refinement, with sample sizes expanding from small case reports to 500-patient cohorts [41]. Across studies, robotic platforms (Da Vinci Xi and SP) have enabled precise peritoneal flap harvest, vaginal canal creation, and improved visualization of pelvic structures. Collectively, the literature suggests that robotic assistance has become an integral adjunct in contemporary gender-affirming vaginoplasty; however, most available evidence remains observational, underscoring the need for standardized outcome reporting and higher-quality comparative studies.

### 3.8. The Promise of AI in Robotic Surgery

Recent advances in artificial intelligence (AI) have expanded the role of surgical robots beyond simply replicating the surgeon’s hand movements. Modern systems can learn, adapt, and provide real-time assistance during operations. As these technologies mature, robotic platforms may evolve toward more collaborative roles by integrating visual, tactile, and kinematic inputs with patient-specific data. However, the extent to which such systems can reliably anticipate complications, reduce errors, or meaningfully augment surgical capability remains an area of active investigation and will require careful validation before broader clinical integration [42,43].

AI has the potential to merge computational power with human expertise. These capabilities can enhance preoperative planning, improve surgical precision, and provide real-time intraoperative decision support. Applications include simulation-based training, quality monitoring, benchmarking to support key performance indicators, continuous learning and improvement, event and outcome prediction, complication management, and even surgeon credentialing [44].

### 3.9. Path Toward Autonomous Surgical Robotics

The convergence of AI and robotic surgery paves the way for autonomous completion of advanced procedures. This requires accurate perception of the surgical situation through synthesis of computer vision and sensorized data, ultimately advancing to adaptive, intelligent decision-making [45].

### 3.10. Training and the Learning Curve

Training in robot-assisted surgery is typically supported through a combination of structured courses, virtual reality-based simulators, and supervised clinical experience. Emerging applications of machine learning and video-based feedback have been proposed as adjuncts to surgical education and may help facilitate skill acquisition, although their effectiveness continues to be evaluated.

A fundamental distinction between open and robot-assisted surgery is the absence of direct tactile feedback. Replicating meaningful haptic sensation requires the integration of high-resolution sensors, robust algorithms to interpret tactile data, and reliable control systems capable of approximating the human sensorimotor loop. While progress has been made, with some platforms incorporating elements of real-time tactile perception to assist with grasp control and instrument handling, these technologies remain under active development and have yet to be fully validated in routine clinical practice [46,47].

### 3.11. Soft Robotics and Future Directions

Soft robotics, which uses materials such as fluids, gels, and elastomers that are functionally closer to humans, offers exciting possibilities. These systems are safer for clinical application, reduce mechanical complexity, and adapt to complex working environments. Soft continuum robots—small, flexible, and strong—can navigate curved pathways to reach difficult-to-access surgical sites, performing tasks with exceptional dexterity [48,49]. Of note to our knowledge, soft robotic technologies have not yet achieved routine clinical implementation; however, they represent a rapidly evolving area of research with substantial technological progress.

Looking ahead, the integration of soft robotics into surgical practice has the potential to revolutionize minimally invasive techniques by combining the safety of compliant structures with the precision of robotic control. Emerging advances include hybrid systems, where rigid robotic arms provide stability while soft, continuum segments perform fine manipulations in delicate regions such as the oropharynx, skull base, or around critical neurovascular structures. Additionally, developments in smart materials and embedded sensing technologies may allow soft robotic instruments to provide real-time haptic feedback, enabling surgeons to “feel” tissue interactions remotely—an element largely missing in current robotic platforms. These innovations hold promise not only for expanding the indications of transoral and craniofacial surgery but also for improving patient outcomes through shorter operative times, reduced adjacent tissue trauma, and enhanced functional recovery.

## 4. Current Limitations and Challenges

Although robotic platforms are designed to facilitate complex surgical tasks, their adoption is associated with a learning curve that may initially affect operative efficiency. Early experiences have reported longer operative times compared with conventional approaches, particularly during the adoption phase. While published studies reflect growing interest in robotic surgery, the available evidence remains mostly low-level evidence as per ASPS grading. The majority of published studies consist of case reports, small case series, and retrospective cohort studies (ASPS Levels III–V), with relatively few comparative analyses and a near absence of randomized or prospective trials. As a result, conclusions regarding the clinical benefit, safety, and cost-effectiveness of robotic assistance must be interpreted with caution. The heterogeneity in study design, patient selection, procedural indications, and outcome reporting further limits the ability to draw definitive comparisons across techniques or platforms.

Current limitations include technological constraints, particularly in complex surgical cases, where current robotic systems may not yet match the adaptability and versatility of conventional techniques [21,23,24]. For example, the absence of tactile feedback may lead to excessive force on delicate structures, thereby increasing the risk of injury. Furthermore, it limits the concomitant two-team approach in complex multisite surgery, which may add operating time to the surgery and decrease the workflow in the operating room. In addition, while robotic-assisted procedures are currently associated with higher upfront costs compared with traditional techniques, their true economic impact remains incompletely defined. Well-designed studies with larger sample sizes and extended follow-up are needed to evaluate potential downstream cost benefits, particularly those related to reductions in postoperative abdominal wall morbidity, such as hernia formation and muscle dysfunction, which may otherwise necessitate additional interventions.

Looking ahead, overcoming these challenges will require collaboration among surgeons, engineers, industry leaders, and policymakers. Technical solutions such as enhanced haptic feedback, smaller and more flexible robotic instruments, and integration of artificial intelligence are under development, but their safe clinical adoption will demand rigorous validation through high-quality multicenter trials. From a systems perspective, addressing cost-effectiveness and equitable access will be critical if robotic surgery is to move beyond high-resource centers and benefit patients globally. Finally, standardized training curricula and credentialing pathways must be established to ensure surgeons worldwide can adopt robotic techniques safely and efficiently, ultimately maximizing the impact of this rapidly advancing technology.

Given the narrative design of this study, it relies on the authors’ interpretation and synthesis of the available evidence. To maintain a clinical and procedural focus, educational- and training-focused studies were excluded, which may limit representation of the broader robotic learning ecosystem. Comparative evaluation of clinical outcomes should be better addressed through procedure-specific systematic reviews with standardized outcome reporting.

## 5. Conclusions

Robot-assisted reconstructive surgery has advanced from a novel concept to a rapidly growing field defined by innovation and precision. Multi-port and single-port systems have enabled the first wave of robotic plastic surgery applications. Early-generation microsurgical robotic platforms are experiencing growing clinical adoption. Continued collaboration and accumulation of real-world data will further refine indications, techniques, and outcomes. Robotic reconstruction is in its early phase yet firmly positioned as a lasting component of modern reconstructive surgery.

## Figures and Tables

**Table 2:** Representative studies describing robotic-prosthesis-based breast reconstruction.

S.N	Author, Year	Study Design	LOE	Country	Procedure Type	Number of Patients	Indication	Robot Used	Application/Role of Robot
1	Lai et al., 2020	Case–control	3	Taiwan	NSM with immediate implant-based reconstruction	40	Breast Reconstruction	Da Vinci	Dissection/mastectomy + IPBR
2	Jeon et al., 2021	Case series	4	Korea	Mastectomy, direct-to-implant reconstruction	16	Breast reconstruction	Da Vinci Xi	Mastectomy + IPBR
3	Joo et al., 2021	Case series	4	Korea	Mastectomy, direct-to-implant reconstruction	2	Breast Reconstruction	Da Vinci SP	Mastectomy + IPBR
4	Kijima et al., 2025	Case report	5	Japan	NSM with implant-based reconstruction	1	Breast Reconstruction	Da Vinci	NSM + IPBR
5	Kim et al., 2025 [24]	Cohort Study	3	Korea	Implant based breast reconstruction	49	Breast reconstruction	Da Vinci Xi and SP	Implant based breast reconstruction

S.N—serial number; NSM—nipple-sparing mastectomy; IPBR—immediate prosthetic breast reconstruction; SP—single-port; Xi—multi-port system; LOE—American Society of Plastic Surgery Level of Evidence.

**Table 3:** Representative studies describing the use of the Robotic microscope.

S.N	Author, Year	Study Design	LOE	Country	Procedure/Flap Type	Number of Patients	Indication	Robot Used	Application/Role of Robot
1	Dermietzel et al., 2022 [25]	Cohort	3	Germany	PAP flap, DIEP	5	Breast reconstruction	RoboticScope	Visualizing anastomosis
2	Chung et al., 2023 [26]	Case Report	5	Korea	LVA	1	Lymphedema	RoboticScope	Visualizing anastomosis
3	De Virgilio et al., 2024	Case Report	5	Italy	Free fibula flap	1	Oral squamous cell carcinoma	RoboticScope	Visualizing anastomosis
4	Mokhtar et al., 2025	Cohort	3	United Arab Emirates	Palatoplasty	4	Cleft palate/lip	RoboticScope	Dissection and visualization

S.N—serial number, PAP—profunda artery perforator flap; DIEP—deep inferior epigastric perforator; LVA—lymphaticovenular anastomosis; LOE—American Society of Plastic Surgery Level of Evidence.

**Table 5:** Representative studies reporting robotic applications in craniofacial and head and neck reconstructive surgery.

S.N	Author, Year	Study Design	LOE	Country	Procedure Type	Number of Patients	Indication	Robot Used	Application/Role of Robot
1	Garfein et al., 2011 [32]	Case report	5	USA	RFFF	1	Squamous cell carcinoma of base of tongue	Da Vinci	Flap inset
2	Bonawitz and Duvvuri, 2012	Case Report	5	USA	ALT flap (2), RFFF (2)	4	TORS for malignant tumor	NR	Oral reconstruction, vessel anastomosis
3	Song et al., 2013	Cohort Study	3	Korea	TORS with RFFF, ALT	5	Head and neck reconstruction	Da Vinci S	TORS tumor dissection, flap inset
4	Bonawitz and Duvvuri, 2013	Case series	4	USA	FAMM flap	5	TORS for malignant tumor	NR	Tumor resection
5	Duvvuri et al., 2013	Retrospective review	3	USA	TORS/Base of Tongue with epiglottoplasty	12	Malignant neoplasm, post-surgical VPI, velopharyngeal stenosis	NR	Flap harvest
6	Hans et al., 2013	Case report	5	France	TORS	2	hypopharyngeal carcinoma	Da Vinci	Flap harvest
7	Lin et al., 2016	Feasibility Study	N/A	China	Mandibular angle split osteotomy	5	Prominent mandibular angle	Unnamed, surgical robotic arm and AR system	Positioning
8	Kayhan et al., 2016 [33]	Cohort	3	Turkey	TORS/base of tongue with epiglottoplasty	25	OSA	Da Vinci	Base of tongue reduction, epiglottoplasty
9	Nadjmi, 2016	Case Series	4	Iran	TORS	10	Cleft Palate	Da Vinci	Palate muscle dissection
10	Biron et al., 2017	Case series	4	Canada	RFFF	18	TORS for oropharyngeal squamous cell carcinoma	Da Vinci S	Tumor resection
11	Lin et al., 2021	Case series	4	China	Genioplasty	6	Asymmetry, dysplasia, overdevelopment	CPSR-I system	Positioning and surgeon force perception
12	Lin et al., 2021	Randomized Controlled Trial	2	China	Genioplasty, mandibular angle osteotomy	15	Craniofacial disease	Unnamed	Osteotomy navigation
13	Zhang et al. 2023 [34]	Clinical study	2	China	MDO	4	HFM	Aurora V3, NDI	Distraction Osteogenesis/Intraoperative Guidance
14	Ebeling et al., 2023	Case Report	5	USA	Le Fort I osteotomy	1	Skeletal class III malocclusion	CARLO	Linear laser osteotomy
15	Porcuna et al., 2023	Cohort Study	3	Spain	TORS/tracheostomy and resection with free flap reconstruction (ALT/RFFF)	15	Oropharyngeal squamous cell carcinoma	Da Vinci Xi	Dissection, vessel exposure, flap inset
16	Li et al., 2025	Cohort	3	China	Mandibular osteotomy	42	Cosmetic, hemifacial microsomia	NR	Mandibular osteotomy, distraction osteogenesis

S.N: Serial Number; ALT—anterolateral thigh flap; RFFF—radial forearm free flap; TORS—transoral robotic surgery; FAMM—facial artery musculomucosal flap; MDO—mandibular distraction osteogenesis; AR—augmented reality; CPSR-I—craniofacial-plastic surgical robot, version i; CARLO—cold ablation robot-guided laser osteotome; HFM—hemifacial microsomia; OSA—obstructive sleep apnea; VPI—velopharyngeal insufficiency; LOE—level of evidence; NR—not reported; NA: not applicable. Numbers in brackets indicate the total number of flaps for each procedure.

**Table 6:** Representative studies describing robot-assisted esthetic procedures. LOE: American Society of Plastic Surgery Level of Evidence.

S.N	Year	Author, Year	Study Design	LOE	Country	Procedure Type	Number of Patients	Indication	Robot Used	Application/Role of Robot
1	2016	Bernstein et al., 2016 [37]	Case Series	4	USA	FUE hair transplant	24	Follicular unit graft selection	ARTAS	Graft harvest
2	2021	Kanayama et al., 2021 [38]	Cohort	3	Japan	FUE hair transplant	31	Alopecia	ARTAS	Follicular harvest
3	2023	Rybakin et al., 2023	Cohort	3	Russia	Rhytidectomy	5	Facial Rejuvenation	Da Vinci Si	Dissection
4	2024	Borisenko et al., 2024 [39]	Case Report	5	Russia	Esthetic lipoabdominoplasty, cholecystectomy	1	Diastasis of the rectus abdominis muscles, cholelithiasis, calculous cholecystits	NR	Dissection, cholecystectomy

S.N—serial number; FUE—follicular unit extraction; NR—not reported. LOE—American Society of Plastic Surgery Level of Evidence.

**Table 7:** Representative studies describing robot-assisted gender-affirming genital surgery.

S.N	Author, Year	Study Design	LOE	Country	Procedure Type	Number of Patients	Indication	Robot Used	Application/Role of Robot
1	Boztosun and Olgan, 2016 [40]	Case Report	5	Turkey	Sigmoid vaginoplasty	1	Mayer-Rokitansky-Kuster-Hauser Syndrome	Da Vinci Xi	Dissection of sigmoid colon graft
2	Jacaby et al., 2019	Retrospective review	3	USA	Vaginoplasty	41	Gender-affirming surgery	Da Vinci	Flap dissection
3	Acar et al. 2020	Case series	4	USA	Vaginoplasty	11	Gender-affirming surgery	Da Vinci Xi, Da Vinci SP	Peritoneal flap harvest and suturing
4	Oriana et al., 2020	Case Report	5	USA	Vaginectomy	16	Gender-affirming surgery	NR	Robotic asissted vaginectomy and muscle harvest
5	Dy et al., 2021	Retrospective review	3	USA	Peritoneal flap revision vaginoplasty	24	Gender-affirming surgery	Da Vinci Xi, Da Vinci SP	Flap harvest and inset
6	Jun et al., 2021	Retrospective review	3	USA	Vaginectomy	42	Gender-affirming surgery	Da Vinci Xi, Da Vinci SP	Flap dissection
7	Dy et al., 2022	Retrospective review	3	USA	Peritoneal flap vaginoplasty	145	Gender-affirming surgery	Da Vinci Xi, Da Vinci SP	Flap harvest and inset
8	Blasdel et al., 2023	Case series	4	USA	Vaginoplasty	43	Gender-affirming surgery	Da Vinci SP	Vaginal canal dissection and peritoneal flap creation
9	Corral et al., 2024	Case series	4	USA	Vaginoplasty	6	Gender-affirming surgery	NR	Flap harvest
10	Blasdel et al., 2025 [41]	Case series	4	USA	Vaginoplasty	500	Gender-affirming surgery	Da Vinci Xi, Da Vinci SP	NR

S.N—serial number; NR—Not Reported; LOE—American Society of Plastic Surgery Level of Evidence.

## Data Availability

Can be provided on request to the corresponding authors.

## Supplementary Materials

The following supporting information can be downloaded at: https://www.mdpi.com/article/10.3390/jcm15020602/s1, Figure S1: Flow diagram illustrating the literature identification and selection process for this narrative review.

## Abbreviations

AI	Artificial Intelligence
ALT	Anterolateral Thigh
DIEP	Deep Inferior Epigastric Perforator
FUE	Follicular Unit Extraction
LVA	Lymphaticovenular Anastomosis
NSM	Nipple-Sparing Mastectomy
OSA	Obstructive Sleep Apnea
SP	Single-Port
TEP	Totally Extraperitoneal
TORS	Trans-Oral Robotic Surgery
VR	Virtual Reality
