# Supplementary material for: Robotic Horizons in Plastic Surgery: A Look Toward the Future
Source: J Clin Med. 2026 Jan 12;15(2):602. doi: 10.3390/jcm15020602 (PMC12841632; doi:10.3390/jcm15020602)
Supplement: Supplementary file 1 [file jcm-15-00602-s001.zip › jcm-4004217-supplementary.pdf]

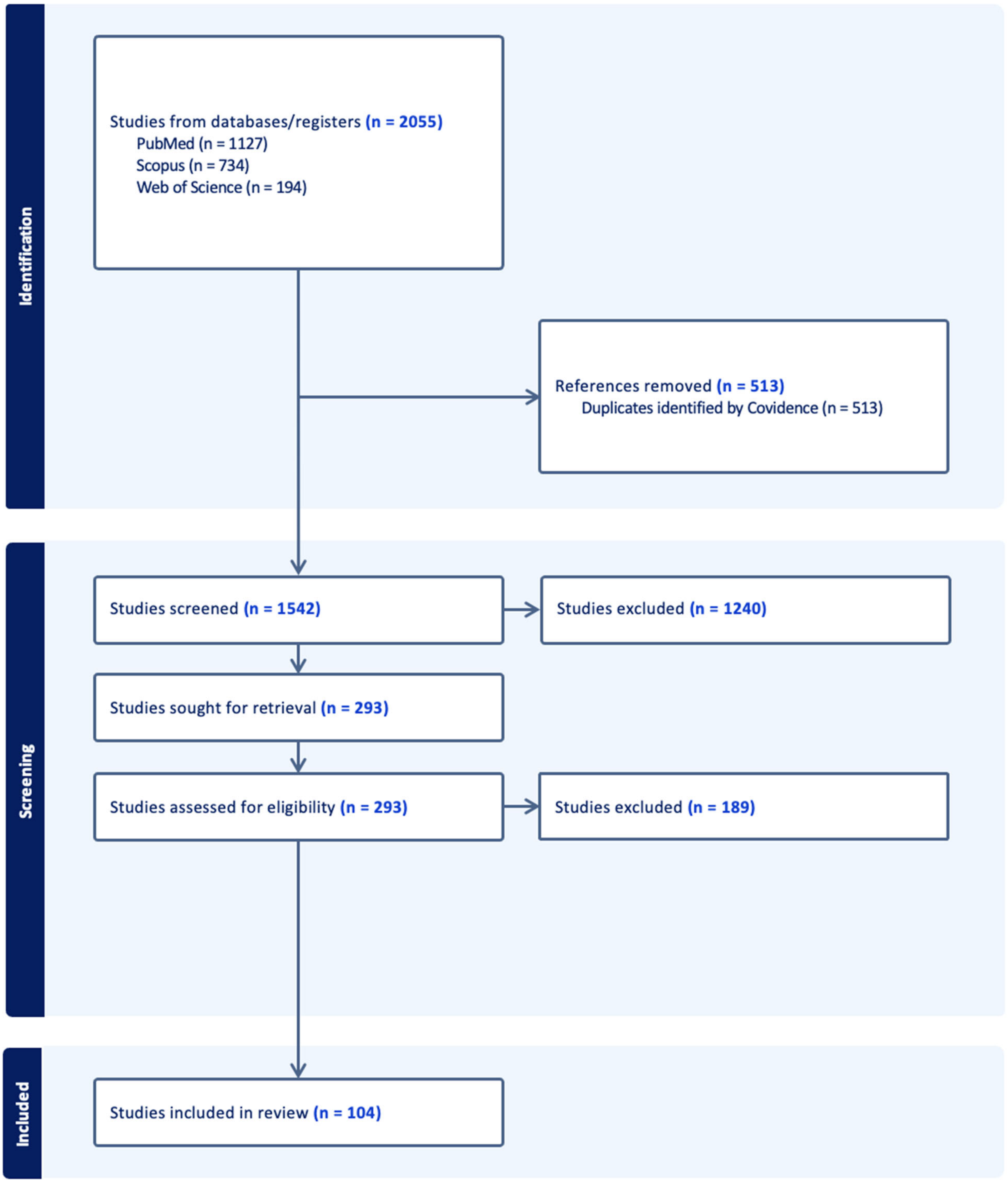

Supplementary Figure S1: Flow diagram illustrating the literature identification and selection process for this narrative review.

Search Terms

Pubmed Searchx

("surgery, plastic"[MeSH Terms] OR ("surgery"[All Fields] AND "plastic"[All Fields]) OR "plastic surgery"[All Fields] OR ("plastic"[All Fields] AND "surgery"[All Fields]))

AND

("robotic surgical procedures"[MeSH Terms] OR ("robotic"[All Fields] AND "surgical"[All Fields] AND "procedures"[All Fields]) OR "robotic surgical procedures"[All Fields] OR ("robotic"[All Fields] AND "surgery"[All Fields]) OR "robotic surgery"[All Fields])
